# The Maternally Inheritable *Wolbachia w*AlbB Induces Refractoriness to *Plasmodium berghei* in *Anopheles stephensi*

**DOI:** 10.3389/fmicb.2017.00366

**Published:** 2017-03-08

**Authors:** Deepak Joshi, Xiaoling Pan, Michael J. McFadden, David Bevins, Xiao Liang, Peng Lu, Suzanne Thiem, Zhiyong Xi

**Affiliations:** ^1^Department of Microbiology and Molecular Genetics, Michigan State UniversityEast Lansing, MI, USA; ^2^Comparative Medicine and Integrative Biology Program, Michigan State UniversityEast Lansing, MI, USA; ^3^Department of Entomology, Michigan State UniversityEast Lansing, MI, USA; ^4^Sun Yat-sen University—Michigan State University Joint Center of Vector Control for Tropical DiseasesGuangzhou, China

**Keywords:** *Wolbachia*, *Plasmodium*, malaria, population replacement, vector-borne disease, innate immunity

## Abstract

The endosymbiont *Wolbachia w*AlbB induces refractoriness to *Plasmodium falciparum* in *Anopheles stephensi*, the primary mosquito vector of human malaria in the Middle East and South Asia. However, it remains unknown whether such refractoriness can be extended to other malaria species. In particular, it was reported that under very specific conditions, *w*AlbB can enhance *Plasmodium* infection in some hosts. Here, we measured the impact of *w*AlbB on the rodent malaria parasite *Plasmodium berghei* in *A. stephensi* by comparing the load of oocysts and sporozoites in midguts and salivary glands, respectively, between *w*AlbB-infected and -uninfected mosquitoes. To investigate whether *w*AlbB modulated mosquito immune defense against parasites, we compared the expression of the immune genes, which were previously reported to involve in antimalarial response, in both midguts and the remaining carcass tissues of mosquitoes. The stable association of *w*AlbB with *A. stephensi* resulted in reduction of parasites by more than half at the oocyst stage, and up to 91.8% at the sporzoite stage. The anti-*plasmodium* immune genes, including *TEP1*, *LRIM1*, Toll pathway gene *Rel1* and the effector *Defensin 1*, were induced by *w*AlbB in different mosquito body tissues. These findings suggest that immune priming is a potential cause of *w*AlbB-mediated antimalarial response in *A. stephensi*. More importantly, no evidence was found for any enhancement of *Plasmodium* infection in *A. stephensi* stably infected with *w*AlbB. We discuss these findings with possible implementations of *Wolbachia* for malaria control in disease endemic areas.

## Introduction

Transmitted by *Anopheles* mosquitoes, malaria is one of the world’s deadliest diseases caused by protozoan parasites of the genus *Plasmodium*. Although significant efforts and resources have been devoted to malaria control, especially over the past decade, there are still 3.2 billion people currently living in areas of high malaria risk, with about 214 million cases of clinical malaria and 438,000 malaria-related deaths in WHO (2015). Given the lack of a highly effective vaccine and the development of drug resistance in parasites and insecticide resistance in mosquito vectors, there is an urgent need for novel control strategies to target the vectors that are difficult to control by the existing methods (The malERA Consultative Group on Vector Control, 2011). One of the potential approaches is to suppress or modify mosquito population using either genetically modified techniques or the endosymbiotic bacterium *Wolbachia* (Ito et al., 2002; The malERA Consultative Group on Vector Control, 2011), which was recently tested for proof-of-concept through field release to control mosquito-borne diseases (Hoffmann et al., 2011; Carvalho et al., 2015; Mains et al., 2016).

*Wolbachia* spp. are intracellular alpha-proteobacteria closely related to Rickettsia. Maternally inherited infections with *Wolbachia* occur in an estimated 40–66% of insect species, and approximately 28% of surveyed mosquito species (Kittayapong et al., 2000; Ricci et al., 2002; Hilgenboecker et al., 2008; Zug and Hammerstein, 2012). No native *Wolbachia* had been found in the 38 surveyed *Anopheles* species until a recent report identified a novel *Wolbachia* strain, related to but distinct from strains infecting other arthropods, in *Anopheles gambiae* (Kittayapong et al., 2000; Baldini et al., 2014; Bourtzis et al., 2014). Through the cytoplasmic incompatibility (CI) mechanism, some *Wolbachia* strains induce early embryonic death when a *Wolbachia*-infected male mates with an uninfected female (Sinkins, 2004; Werren et al., 2008). Thus, *Wolbachia*-infected male mosquitoes are proposed to be released to induce incompatible (or sterile) mating and reduce vector density below a threshold required for disease transmission (Laven, 1967; Brelsfoard et al., 2008). Because *Wolbachia*-infected females produce viable offspring whether they mate with uninfected or infected males, CI provides the infected females an advantage in reproduction over the uninfected female, allowing *Wolbachia* to spread into an uninfected population. With the ability of *Wolbachia* to directly reduce malaria parasites in the *Anopheles* mosquito (Bian et al., 2013), invasion of *Wolbachia* into vector populations theoretically will reduce mosquito vector competence for malaria parasites, resulting in intervention of disease transmission. The feasibility of the above two strategies in disease control is currently being tested to combat Zika and dengue through field trials in many countries, including Australia, USA, China and Brazil, using *Aedes* mosquitoes carrying different type of *Wolbachia* (Hoffmann et al., 2011; Bourtzis et al., 2014; Xi and Joshi, 2015; Dutra et al., 2016; Mains et al., 2016).

We previously generated the first maternally inheritable *Wolbachia* infection in an *Anopheles* malaria vector by transferring *Wolbachia w*AlbB from *Aedes albopictus* into *A. stephensi* through embryonic microinjection, resulting in establishment of the transinfected LB1 strain in 2011 (Bian et al., 2013). Since then, *w*AlbB has been stably maintained in the *A. stephensi* LB1 strain in the laboratory for more than 5 years, displaying both perfect maternal transmission and the ability to induce a nearly complete CI. Furthermore, *w*AlbB induces refractoriness to the human malaria parasite *Plasmodium falciparum* in the LB1 mosquito (Bian et al., 2013). This is consistent with the observation that a stable *w*MelPop infection reduced the infection of avian malaria parasite, *P. gallinaceum*, in *Aedes aegypti* (Moreira et al., 2009). Without a stable infection in *Anopheles gambiae*, a transient *Wolbachia* infection was used as a model to study *Wolbachia*–*Plasmodium* interactions in this mosquito species (Kambris et al., 2010; Hughes et al., 2011). This transient infection differed from a stable infection in that it was generated through adult intrathoracic injection and the infection was lost in the subsequent generations. Consistently, a transient *w*MelPop infection significantly reduced both *P. falciparum* and *Plasmodium berghei* in *A. gambiae* (Kambris et al., 2010; Hughes et al., 2011). Suppression of *P. falciparum* was also observed in *A. gambiae* with a transient *w*AlbB infection (Hughes et al., 2011). In addition, the observed reduction was associated with induction of anti-*Plasmodium* immune genes, including *TEP1* (Kambris et al., 2010).

However, the impact of *Wolbachia* on *Plasmodium* in mosquito may differ, depending on the strain of *Wolbachia*, the species of parasite and the environmental temperature. In contrast to the pathogen interference described above, a native *w*Pip infection was claimed to enhance the avian malaria parasite *P. relictum* in *Culex pipiens* mosquito (Zele et al., 2014). A transient *w*AlbB infection was also reported to increase the rodent parasite *P. berghei* oocyst load in the midgut of *A. gamibiae*, and the *P. yoelii* oocyst load at 24°C in *A. stephensi* (Hughes et al., 2012; Murdock et al., 2014). It is known that *P. falciparum* and *P. berghei* interact differently with mosquito hosts and the other four human malaria species, *P. malariae, P. ovale, P. knowlesi*, and *P. vivax* are phylogenetically more closely related to *P. berghei* than they are to *P. falciparum* (Hughes et al., 2014). This raises concerns whether *Wolbachia*-based population replacement may enhance transmission of those parasites (Hughes et al., 2014). Thus, it is essential to validate the impact of a stable *w*AlbB infection on mosquito vector competence for additional *Plasmodium* species in *Anopheles* mosquitoes.

In this study, we compared the vector competence for *P. berghei* between of *Wolbachia*-infected and -uninfected *A. stephensi* by examining oocyst and sporozoite loads in mosquito midguts and salivary glands, respectively. To confirm whether *Wolbachia* was able to regulate expression of the host immune genes, we compared the expression of several immune genes, which are known to be involved in antimalarial response, in both midguts and the remaining carcass tissues between the LB1 strain and *Wolbachia*-uninfected *A. stephensi*. We observed that *w*AlbB significantly reduced *P. berghei* at both oocyst and sporozoite stage in LB1 mosquitoes. This reduction was associated with induction of a number of anti-*Plasmodium* immune genes, including *TEP1*, *Rel1* and *Defensin 1*, in either midgut or the carcass. Consistent with our previous studies using blood-fed mosquitoes, there was no impact of *w*AlbB on mosquito life span after taking *P. berghei*-infected blood meal.

## Materials and Methods

### Mosquito Rearing

The wild-type *A. stephensi* LIS strain (*Wolbachia*-free), *A. stephensi* LB1 strain (*Wolbachia*-infected) and the aposymbiotic line LBT strain (*Wolbachia*-free; generated by tetracycline treatment of the LB1 strain to remove *w*AlbB) were described previously (Bian et al., 2013) and maintained on sugar solution at 27°C and 85% humidity, with a 12-h/12-h light/dark cycle, according to standard rearing procedures (Joshi et al., 2014). Before the infection assay, females of both LBT and LB1 were outcrossed with LIS males for >4 generations. For colony maintenance and *Plasmodium* infection assay, adult females were fed on the blood of anesthetized mice (BALB/c) according to a protocol (03/14-036-00) approved by Michigan State University Institutional Animal Care and Use Committees.

### *P. berghei* Infection Assay

*Plasmodium berghei* (ANKA 2.34 strain) parasites from frozen stocks were administered intraperitoneally to donor mice. When the parasitemias of donor mice reached 10–20%, infected blood was collected by heart puncture, washed and diluted 2.5 times with PBS. Then, 200 μl of this diluted blood was transferred to naive mice via intraperitoneal injection. All mice were 4- to 6-week-old BALB/c females. Parasitemia and exflagellation rates were assessed by light microscopy inspection of Giemsa-stained thin smears obtained by tail snips before mice were used for mosquito feeding. At 7–8 days post-emergence, 60–70 females were transferred into a 0.5-lt mesh-covered cardboard cup and were deprived of sucrose solution for 1–2 h, then allowed to feed on anesthetized mice infected with *P. berghei* that exhibited 1–3 exflagellation events per field, as previously described (Billker et al., 1997). All *P. berghei*-infected mosquitoes and corresponding control mosquitoes were kept at 21°C and 80% humidity. At 10 days post-blood feeding, mosquito midguts were dissected and stained in 0.05% mercurochrome for at least 10–30 min, and the loads of *P. berghei* oocysts were quantified under light microscopy at 10X magnification. At 21 days post-infection, mosquito salivary glands were dissected to quantify the infection intensity at the stage of sporzoite. The salivary glands were transferred into a microfuge tube containing 120 μl of PBS and homogenized gently for 30 s with a hand held pestle. After centrifugation at 8,000 rpm for 10 min, 90 μl of the supernatant was discarded. The sporozoites were resuspended in a final volume of 30 μl of PBS, and 10 μl of this suspension was used to count the sporozoites as described previously (Bian et al., 2013).

### RNA Extraction, cDNA Synthesis, and qRT PCR

Seven- to nine-day-old non-blood fed females were dissected in PBS and midguts and the remaining carcass tissues were collected in TRIzol^®^ reagent (Life technologies) and stored at -80°C until RNA extraction. Each tissue had eight replicates, with samples of five females from a cohort of mosquitoes pooled together to make one replicate. The cDNA transcript was produced using Reverse Transcription Kit (Invitrogen). Real time PCR was performed using SYBR green kit (Qiagen sciences) and ABI Prism 7900HT Sequence Detection System (Applied Biosystems). The data were processed and analyzed with Applied Biosystems SDS2.3 software and the obtained CT values were exported into the Excel program to calculate the relative fold changes. The ribosomal protein S6 (RPS6) was used as an internal control for normalization of cDNA templates (Bian et al., 2013). Relative fold changes in gene expression values between *Wolbachia*-infected (LB1) and uninfected (LBT) tissues were obtained by using 2^-ΔΔ^*^C^*^T^ method (Livak and Schmittgen, 2001). All primers used for real-time PCR are listed in Supplementary Table S1.

### Life Span Assays

After feeding on *P. berghei*-infected mice, LB1, LIS, and LBT mosquitoes were sorted and transferred into 0.5-lt mesh-covered cardboard cups, with at least 20 females for each of 2–4 replicates. Cups were changed every 2 days to avoid the impact of fungi, which may grow on the dead mosquitoes, on the data. Mortality was recorded on daily basis until day 9 post-infection, when over 70% of death had occurred.

### Statistical Analyses

Prior to analyses, the normality of the data sets was checked using D’Agostino and Pearson omnibus normality test. The oocyst and sprozoite data were not normally distributed. Thus, the Mann–Whittney *U*-test based on median values were used for analysis. The prevalence rates in mosquitoes, in terms of midgut and salivary gland infection rates, were analyzed using Fisher’s exact test and the expression values of the genes were analyzed using Student’s *t*-test. Log-rank test was used to compare the survivorship after mosquitoes took *P. berghei*-infected blood. All the analysis was done using GraphPad Prism version 5.00.

## Results

### *w*AlbB Reduces *P. berghei* Oocyst Loads in Mosquito Midguts

**FIGURE 1:**
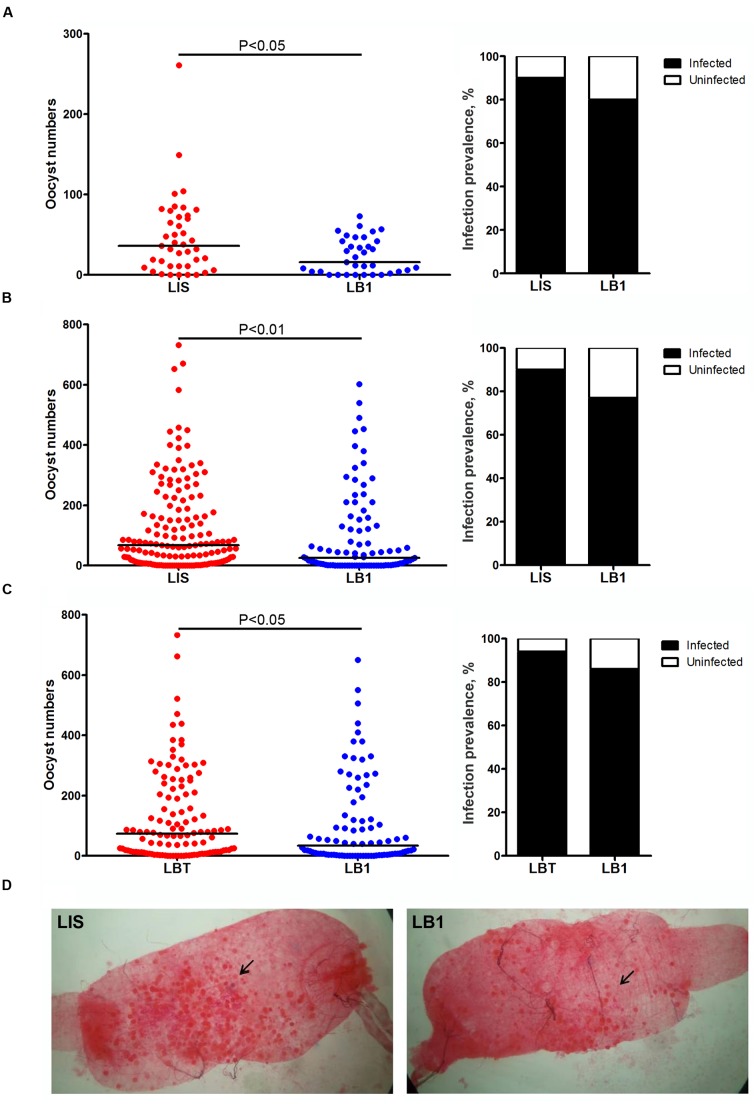
***Wolbachia w*AlbB-mediated reduction in *Plasmodium berghei* oocyst load in LB1 midguts**. *P. berghei* oocyst loads in midguts of *Anophelus stephensi* LB1 strain and its infection prevalence (the percentage of mosquitoes that were infected at any level) are compared to those of LIS **(A,B)** and LBT strains **(C)**. Points represent the number of parasites from an individual mosquito and horizontal lines indicate the median number of parasites per tissue. *P*-value is indicated based on Mann–Whitney test. **(D)** One representative midgut from LIS and LB1 strain is shown, with an arrow indicating the oocyst.

We previously showed that a stable *w*AlbB infection reduced *P. falciparum* oocyst loads in midguts of *A. stephensi* (Bian et al., 2013). However, a transient *w*AlbB infection was reported to increase *P. berghei* oocyst load in midguts of *A. gambiae* (Hughes et al., 2012). In order to characterize the effect of *w*AlbB on vector competence of *A. stephensi* for *P. berghei*, we compared the oocyst levels between the wild-type LIS strain (*Wolbachia*-free) and LIS-derived LB1 strain (*w*AlbB -infected) or between LB1 strain and its aposymbiotic LBT strain at day 10 after they took an infectious blood meal from mice. In all three experiments, LB1 mosquitoes displayed a significant reduction in the oocyst loads, with a trend of reduction in the infection prevalence, compared to the LIS or LBT mosquitoes (**Figure 1**). Specifically, a significant reduction in oocyst load was observed in midguts of LB1 mosquitoes compared to those of LIS mosquitoes in both experiments 1 and 2 (Mann–Whitney *U*-test, *P* < 0.05 and < 0.01 in experiments 1 and 2, respectively) (**Figures 1A,B** and Supplementary Table S2). The median number of oocysts was reduced by 55.6 and 63% in LB1 midguts compared to LIS midguts in experiments 1 and 2, respectively. In experiment 3, a similar experiment was performed to compare oocyst development in LB1 mosquitoes and LBT strain. Consistently, there was a significant reduction in oocyst load in midguts of LB1 mosquitoes compared to those of LBT mosquitoes (Mann–Whitney *U*-test, *P* < 0.05) (**Figure 1C** and Supplementary Table S2). These results indicate that *w*AlbB interferes with *P. berghei* oocyst development in midguts of LB1 mosquitoes.

### *w*AlbB Reduces *P. berghei* Sporozoite Loads in Mosquito Salivary Glands

**FIGURE 2:**
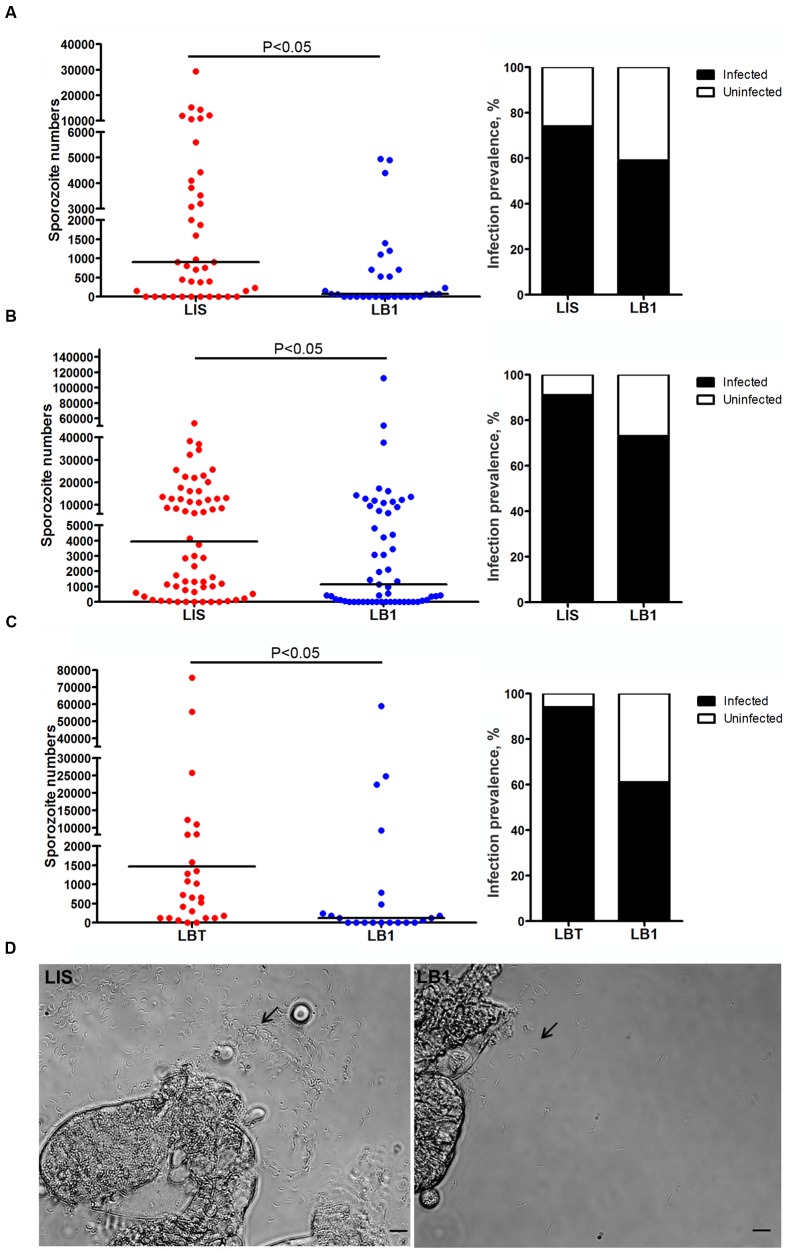
***Wolbachia w*AlbB-mediated reduction in *P. berghei* sporozoite loads in LB1 salivary glands**. *P. berghei* sporozoite loads in salivary glands of *A. stephensi* LB1 strain and its infection prevalence are compared to those of LIS **(A,B)** and LBT strains **(C)**. Points represent the number of parasites from an individual mosquito and horizontal lines indicate the median number of parasites per tissue. *P*-value is indicated based on Mann–Whitney test. **(D)** One representative salivary glands from LIS and LB1 strain is shown, with an arrow indicating the sporozoite.

To further test whether mosquito’s potential to transmit *P. berghei* was reduced by *w*AlbB, we compared the number of sporozoites in the salivary glands of LB1 and LIS or LBT mosquitoes at 21 days post-infection. In all three experiments, there were significantly lower numbers of sporozoites, with a trend of reduction in the infection prevalence, in the salivary glands of LB1 mosquitoes than those of LIS or LBT mosquitoes (Mann–Whitney *U*-test, *P* < 0.05) (**Figure 2** and Supplementary Table S2). In experiments 1 and 2, the median number of sporozoites was reduced by 91.7 and 60.2%, respectively, in LB1 mosquitoes compared to LIS mosquitoes (**Figures 2A,B** and Supplementary Table S2). In experiment 3, the median number of sporozoites was reduced by 91.8% in LB1 mosquitoes compared to LBT mosquitoes (**Figure 2C** and Supplementary Table S2). These results indicate *w*AlbB interferes with *P. berghei* sporozoite development in mosquito salivary glands.

### *w*AlbB Induces Expression of Anti-*Plasmodium* Immune Factors in Mosquito

**FIGURE 3:**
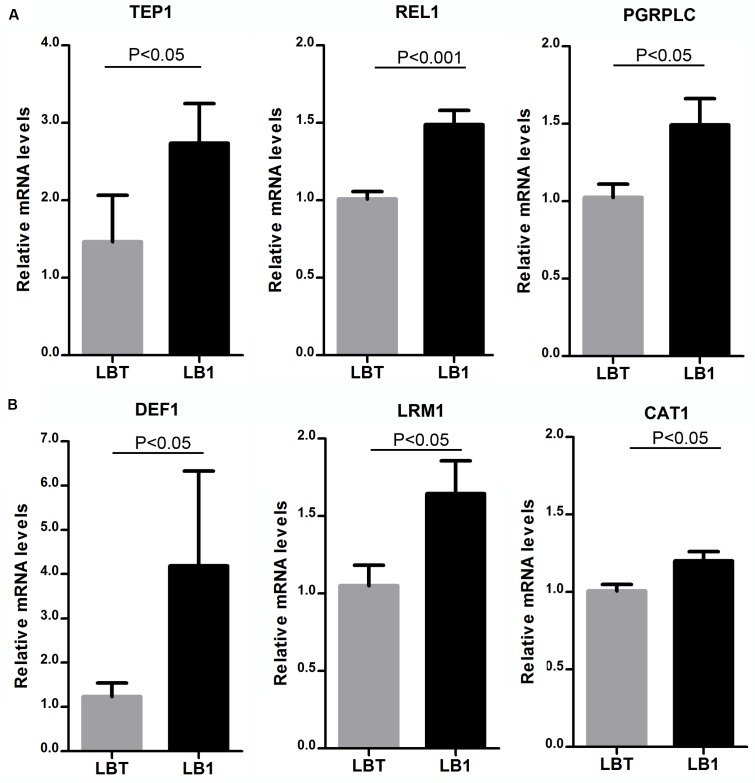
**Up-regulation of anti-*Plasmodium* immune genes by wAlbB in LB1 mosquito**. qRT-PCR was used to assess the expression of each of the selected anti-*Plasmodium* immune genes in either midguts **(A)** or the remaining carcass tissues **(B)**, with the host RPS6 as an internal reference control to normalize the data. 2^-ΔΔCT^ method was used to calculate fold change for each gene and significance was determined based on comparison of ΔCT of each gene in LB1 and LBT. *P*-value is indicated based on Mann–Whitney test.

To explore the potential mechanism underlying *w*AlbB-mediated *Plasmodium* interference, we selected six immune genes that were previously reported to play roles in anti-*Plasmodium* response (Blandin et al., 2004; Frolet et al., 2006; Povelones et al., 2009) and compared their expression in both midguts and the remaining carcass tissues of non-blood-fed mosquito LB1 and LBT strains using qRT-PCR. As a result, we found that expression of *TEP1*, *Rel1*, and *PGRP-LC* genes were significantly induced by *w*AlbB in midguts (**Figure 3A**) and expression of *Def1*, *LRIM1*, and *CAT* genes were significantly induced in the carcass tissues (**Figure 3B**). These results indicate that *w*AlbB may induce parasite interference through priming the mosquito’s anti-*Plasmodium* immune system.

### *w*AlbB Does Not Change the Life Span of *P. berghei*-Infected Mosquitoes

**FIGURE 4:**
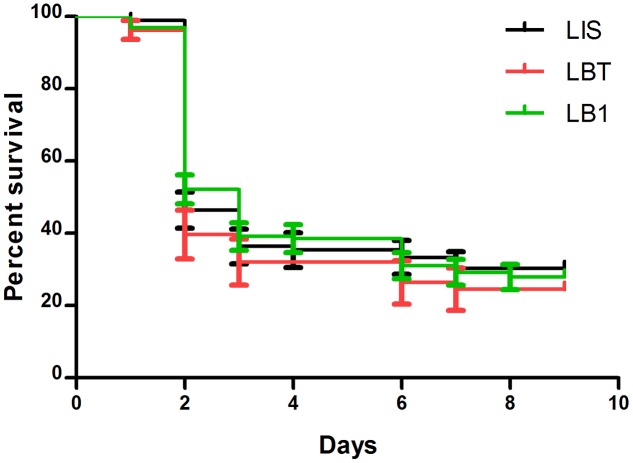
**The impact of *w*AlbB on the life span of LB1 after taking the *P. berghei*-infected blood meal**. After LB1, LIS, and LBT mosquitoes were infected by *P. berghei*, dead mosquitoes were removed and recorded every day. The curves represent the mean percentage of mosquitoes surviving from different biological replicates each day. There was no significant difference in the life span of the infected females between LB1, LIS, and LBT mosquitoes.

We previously observed that *w*AlbB increased longevity of LB1 mosquitoes when they were maintained using 10% sugar solution (Joshi et al., 2014), resulting in a concern because infectious mosquitoes with increased longevity may facilitate disease transmission (Killeen et al., 2013). Thus, we compared the life spans of LB1, LIS, and LBT mosquitoes after they took the *P. berghei*-infected blood meal. All three strains showed a high mortality within 2 days after taking the blood meal and thereafter maintained a low mortality (**Figure 4**). There were no statistical differences in survivorship among LB1, LIS, and LBT mosquitoes (log-rank test, *P* > 0.05). Taken together with the observation of no change in LB1 female longevity after taking uninfected blood (Joshi et al., 2014), our results confirm that *w*AlbB-associated increase in mosquito survivorship will occur only in males or females that have not taken blood.

## Discussion

We have previously shown that *w*AlbB interferes with *P. falciparum* in the *A. stephensi* LB1 strain (Bian et al., 2013). In order to better characterize the spectrum of *w*AlbB-mediated *Plasmodium* interference, we examined the impact of *w*AlbB on *P. berghei* development in LB1 mosquitoes in this study. We found that *w*AlbB reduced both oocyst and sporozoite loads in midguts and salivary glands, respectively, of LB1 mosquitoes. Furthermore, this reduction was associated with up-regulation of a number of anti-*Plasmodium* immune genes, including *TEP1*, *Rel1* and *Def1*, in LB1 mosquitoes. We also showed that *w*AlbB infection did not increase the life span of mosquitoes after taking *P. berghei* -infected blood. These results support the potential to develop *Wolbachia*-based strategy for malaria control.

The strength of *w*AlbB-mediated reduction in *P. berghei* infection is similar to what was previously observed on *P. falciparum* infection (Bian et al., 2013). The oocyst load of both *P. berghei* and *P. falciparum* was reduced by more than half in LB1 midguts compared to LIS midguts. For both parasites, there was stronger reduction in the sporozoite stage than in the oocyst stage. The reduction in *P. berghei* sporozoite levels ranged between 71.1 and 91.8% as shown in the three experiments while *P. falciparum* sporozoite levels were reduced by 70.9% in the previous studies. Thus, these results indicate that *w*AlbB reduces *P. berghei* infection as effectively as *P. falciparum* infection in LB1 mosquitoes.

The ability of *w*AlbB to reduce both *P. berghei* and *P. falciparum* in *A. stephensi* indicates that *w*AlbB may target common host factors that are essential for *Plasmodium* development in mosquitoes. Two main hypotheses have been proposed to understand the mechanism of *Wolbachia*-mediated pathogen interference in insect hosts (Bourtzis et al., 2014; Xi and Joshi, 2015). First, *Wolbachia* primes host immunity such that it can effectively react to inhibit the subsequent pathogen invasion. This is supported by the fact that a number of immune genes, including Toll pathway genes and redox genes, were induced by *Wolbachia* in the transinfected lines (Pan et al., 2012). Second, *Wolbachia* outcompetes pathogens in utilizing host metabolic pathway/components for its intracellular growth. For example, *Wolbachia* replication is cholesterol-dependent, requiring cholesterol-rich host membranes to form the vacuole surrounding each bacterium (Caragata et al., 2013). This could lead to a competition for cholesterol between *Wolbachia* and pathogens (Atella et al., 2009).

To test the first hypothesis, we compared the expression of six immune genes that were reported to be involved in blocking of human and mouse malaria in *A. gambiae* (Blandin et al., 2004; Frolet et al., 2006; Povelones et al., 2009). We found that *TEP1*, *PGRP-LC*, and *REL1* were induced in the midgut while *CAT1*, *Def1*, and *LRIM1* were up-regulated in the carcass. Among them, *TEP1* and *Def1* were induced 2.7- and 4.2-fold, respectively, by *w*AlbB in LB1 mosquitoes. These results are consistent with the previous studies using *A. gambiae* with a transient somatic *w*MelPop infection and a stably infected cell line, in which *w*MelPop strongly induced the expression of *TEP1*, *LRIM1*, and *Def1* (Kambris et al., 2010). Further studies show that a recombinant *Wolbachia* surface protein (WSP) is sufficient to induce the expression of those malaria-related immune genes in both *Anopheles* and *Aedes* cell lines (Pinto et al., 2012).

As a well-characterized complement-like molecule in the anti-parasitic defense of mosquitoes, TEP1 plays a central role in defense against gram negative bacteria, *P. falciparum* and *P. berghei* in *Anopheles* mosquitoes. With the capacity to reduce the oocyst burden by either direct killing or melanization process, TEP1 was described as major determinant of vectorial capacity for *Plasmodium* in *A. gambiae* (Blandin et al., 2004). Knocking down TEP1 gene expression has resulted in several fold increase in the midgut oocyst load (Dong et al., 2006; Frolet et al., 2006). In *A. gambiae*, Leucine-rich Repeat (LRR) proteins, LRIM1, and APL1C, form complexes to facilitate recognition of the parasite by TEP1, resulting in parasite killing either by a lytic mechanism or by arresting their development through melanization (Fraiture et al., 2009; Povelones et al., 2009). Consistently, LRIM1 was observed to be up-regulated by *w*AlbB in the carcass of LB1 mosquitoes. However, the orthologes of the *A. gambiae* sub family APL genes (*APL1-A*, *APL1-B*, and *APL1-C*) are absent in the genome of *A. stephensi* (Jiang et al., 2014). It is unknown whether another molecule plays a role similar to APL1C to form a comparable complex in *A. stephensi*. Evidence has shown that boosting NF-Kappa B factors (REL1 and REL2) induces production of TEP1 in *A. gambiae* (Frolet et al., 2006). We observed that REL1 and IMD pathway gene PGRP-LC were also up-regulated by *w*AlbB in LB1 mosquitoes. Activation of Toll and IMD pathways by *w*AlbB was further supported by a strong up-regulation of expression of the antimicrobial peptide DEF1. DEF1 has a profound effect on development of oocysts and sporozoites. Previous studies have shown that either injection or over-expression of defensin in *A. aegypti* reduced both *Plasmodium* oocyst in midgut and sporozoite in salivary gland (Shahabuddin et al., 1998; Kokoza et al., 2010).

We previously found that the levels of ROS were significantly higher in midguts and fat bodies of LB1 mosquitoes than in those of LIS mosquitoes and nearly twofold higher in the whole body of LB1 than in that of LIS mosquitoes (Bian et al., 2013). Interestingly, midgut epithelial nitration has been shown to work as an opsonization system that promotes activation of the mosquito complement cascade (Kumar et al., 2010). It would be interested to know whether epithelial nitration is increased in *Plasmodium*-infected midguts of LB1 mosquitoes, resulting in enhancement of TEP1-mediated lysis for anti-*Plamsmodial* immunity. Increased production of ROS can lead to melanization of parasites in an *A. gabmbiae* refractory strain (L3 strain) (Kumar et al., 2003). However, we did not observe the deposition of the melanin pigment on the surface of the oocysts in midguts of the LB1 strain.

While we saw that *w*AlbB reduced *P. berghei* at both oocyst and sporozoite stages in LB1 mosquito, in contrast it was reported that *A. gambiae* with a transient *w*AlbB infection enhanced *P. berghei* oocyst load (Hughes et al., 2012). The same group also reported that the number of *P. yoelii* oocyst increased in *A. stephensi* with a transient *w*AlbB infection at 24°C although *w*AlbB reduced the parasite at warmer temperatures (Murdock et al., 2014). We think that they are likely to be artifacts associated with their used transient infection system, in which *Wolbachia* infection cannot pass to mosquito offspring because a barrier appears to block the ability of *Wolbachia* to infect the germ line. Mosquito lines with stable maternal inheritable infections are generated through embryonic microinjection, during which *Wolbachia* is introduced into germ plasm before the germ cells are formed in the early embryo. Because only a small percentage of the surviving individuals will have acquired a germ-line *Wolbachia* infection through this process, a further intensive screen is carried out to identify a stably infected line, which can maternally transmit *Wolbachia* to the next generations at 100% efficiency. Thus, a stable infection system has passed through a restricted selection process that results in both *Wolbachia* and its host being able to adapt to each other and to maintain their co-existence. It is possible that only a subset of the variants in a population of *Wolbachia* have been selected to form the symbiosis in the stable infection system. Some *Wolbachia* may not be able to form symbiosis due to inability to adapt to the new host, which usually occurs when *Wolbachia* is transferred between phylogenetically distant hosts. The requirement for embryonic injection to generate a stable infection in mosquito may indicate that early contact between *Wolbachia* and the host (i.e., earlier than the development of the host immune system) may be important in shaping the host’s immune system so that *Wolbachia* can be persistently maintained in a new host. In a transient infection system, the process of selection, adaptation, symbiosis formation, and shaping of the host immune system is avoided, thus, it can represent a very different type of system than that of a stably infected system.

When evaluating the impact of *Wolbachia* on mosquito vector competence, we think that two gold standards should be used. First, only mosquito with a stable *Wolbachia* infection should be used to provide conclusive evidence on whether pathogen interference will occur. This is because only stably infected mosquito can be developed for implementation in disease control, and as described above, the transient infection system does not accurately mimic the stable infection system. Thus conflicting results can be misleading and damage the public perception about the ongoing field trials and future implementation of this control method. Second, the impact of *Wolbachia* on mosquito vector competence should focus on the pathogen transmission stage. This means direct measurement of the number and infectiousness of pathogens that will be transmitted from mosquito to vertebrate host, such as those in mosquito saliva or salivary gland. When *Plasmodia* enter and develop in the midgut, disseminate through hemolymph, and infect the salivary gland, they will be attacked by *Wolbachia*-mediated interference at each stage because *Wolbachia* has preoccupied those tissues and induced hostile environment either in tissue-specific (e.g., in midgut and salivary gland) or systemic (e.g., in fat body) ways. An accumulated effect will be observed best at the final transmission stage. It is possible that no reduction or even slight enhancement could be observed in the midgut but strong inhibition still occurs in saliva or salivary gland.

*Anopheles stephensi* is an interesting model species for these studies as its delay in producing a peritrophic matrix (PM, chitin/protein matrix that forms around the blood meal in the gut), which is more than twice as long as that of *A. gambiae* (Freyvogel and Staeubli, 1965). This PM barrier delay results in many more motile ookinetes escaping the gut to form oocysts. Presence of these large number of oocysts (>100s) in a mosquito is laboratory effect because in the field (as seen with other *Anopheles* species and human *Plasmodium* sp.) only one or a couple of oocysts are normally found on the midgut in a potentially infectious field collected *Anopheles* (Sinden et al., 2004). Thus a reduction in oocysts by 50% can mean a lot in the field – however as an oocyst can produce over 1,000 sporozoites, it is likely not the number of oocysts that make a mosquito infective. It would be interested to determine the level of inhibition that is sufficient for *Wolbachia* to interfere with disease transmission in the field.

The two stable *Wolbachia*-infected mosquitoes examined to date show resistances to malaria parasites, including *P. falciparum, P. berghei*, and *P. gallinaceum* (Moreira et al., 2009; Bian et al., 2013). It is argued that this resistance occurs only in recent *Wolbachia*-host associations and will disappear during the long-term evolution of novel *Wolbachia*-host associations in mosquitoes. A recent study has discovered a marginally higher number of the avian malaria parasite *P. relictum* in *Culex pipiens* mosquito with native *w*Pip infection as compared to its aposymbiotic strain derived from antibiotic treatment, resulting in a speculation that those transinfected mosquitoes may evolve to be a better malaria vector (Zele et al., 2014). We think that it is too early to make this prediction because many other reasons, such as host body size and genetic background, cannot be excluded to cause the subtle increase observed in that study. In addition, antibiotic treatment to remove *Wolbachia* has a systemic effect on the whole microbiome of an organism and may result in a multitude of effects especially on the immune system and infection responses. Currently, it is difficult to design laboratory experiments to mimic this long-term evolution process. What we know, however, is that *Wolbachia*-mediated pathogen interference has been maintained for >12 years in *A. aegypt* and >5 years in *A. stephensi* in laboratory conditions. Here, we may be able to learn from the Mycobacterium bovis bacille Calmette–Guérin (BCG) vaccine against tuberculosis (TB). Although BCG is protective for only 10–20 years, it has maintained its position as the world’s most widely used vaccine (Andersen and Doherty, 2005). The ability to both generate a better mosquito strain with improved pathogen blocking and repeatedly spread novel *Wolbachia* into population will allow us to develop either a better strain to replace the old one or a method to boost its effectiveness should the pathogen interference decline over time.

## Conclusion

We have shown that a maternal inheritable *w*AlbB infection can reduce *P. berghei* in *A. stephensi*. This pathogen interference is associated with up-regulation of TEP1, LRIM1, REL1 and the other anti-*Plasmodium* immune genes. *w*AlbB has no impact on the longevity of females after taking infected blood meal although it increases male mosquito survivorship (Joshi et al., 2014). Future studies will investigate the contribution of those up-regulated immune genes to the overall effect of *Wolbachia*-mediated *Plasmodium* interference. Understanding of the mechanism of *Wolbachia*-host interactions will facilitate the development of transinfected mosquito strains with strong pathogen blocking, low fitness cost, stable maternal transmission and complete CI expression. Because there are overlaps in distribution of the parasite *P. vivax* and *A. stephensi*, future studies should also characterize the impact of *w*AlbB on vector competence for *P. vivax.* Since the endosymbiotic bacterium *Wolbachia* was introduced into the primary dengue vector, a global effort, with field trials ongoing in eight countries, has been initiated to develop *Wolbachia* for dengue control. Like the primary dengue vector *A. aegypti*, *A. stephensi* is an urban vector, which allows the transfer our experience from the dengue control field trials to malaria control field trials with this mosquito species more likely to succeed. The ability of *w*AlbB to stably infect an *Anopheles* malaria vector, induce a complete CI and confer mosquito resistance to malaria parasite has opened an exciting opportunity to develop *Wolbachia*-based strategy for malaria control.

## Author Contributions

Conceived and designed the experiments: DJ and ZX. Performed the experiments: DJ, XP, MM, DB, XL, and PL. Analyzed the data: DJ and ZX. Contributed reagents/materials/analysis tools: DJ. Wrote the paper: DJ, ST, and ZX.

## Conflict of Interest Statement

ZX is affiliated with Guangzhou Wolbaki Biotech, Co., Ltd. The other authors declare that the research was conducted in the absence of any commercial or financial relationships that could be construed as a potential conflict of interest.

## References

[B1] Andersen P. Doherty T. M. (2005). The success and failure of BCG - implications for a novel tuberculosis vaccine. *Nat. Rev. Microbiol.* 3 656–662. 10.1038/nrmicro1211 16012514

[B2] Atella G. C. Bittencourt-Cunha P. R. Nunes R. D. Shahabuddin M. Silva-Neto M. A. (2009). The major insect lipoprotein is a lipid source to mosquito stages of malaria parasite. *Acta Trop.* 109 159–162. 10.1016/j.actatropica.2008.10.004 19013123

[B3] Baldini F. Segata N. Pompon J. Marcenac P. Robert Shaw W. Dabire R. K. (2014). Evidence of natural *Wolbachia* infections in field populations of *Anopheles gambiae*. *Nat. Commun.* 5:3985 10.1038/ncomms4985 PMC4059924 24905191

[B4] Bian G. Joshi D. Dong Y. Lu P. Zhou G. Pan X. (2013). *Wolbachia* invades *Anopheles stephensi* populations and induces refractoriness to *Plasmodium* infection. *Science* 340 748–751. 10.1126/science.1236192 23661760

[B5] Billker O. Shaw M. K. Margos G. Sinden R. E. (1997). The roles of temperature, pH and mosquito factors as triggers of male and female gametogenesis of *Plasmodium berghei* in vitro. *Parasitology* 115(Pt 1), 1–7. 10.1017/S0031182097008895 9280891

[B6] Blandin S. Shiao S. H. Moita L. F. Janse C. J. Waters A. P. Kafatos F. C. (2004). Complement-like protein TEP1 is a determinant of vectorial capacity in the malaria vector *Anopheles gambiae*. *Cell* 116 661–670. 10.1016/S0092-8674(04)00173-4 15006349

[B7] Bourtzis K. Dobson S. L. Xi Z. Rasgon J. L. Calvitti M. Moreira L. A. (2014). Harnessing mosquito-*Wolbachia* symbiosis for vector and disease control. *Acta Trop.* 132(Suppl.), S150–S163. 10.1016/j.actatropica.2013.11.004 24252486

[B8] Brelsfoard C. L. Sechan Y. Dobson S. L. (2008). Interspecific hybridization yields strategy for South Pacific filariasis vector elimination. *PLoS Negl. Trop. Dis.* 2:e129 10.1371/journal.pntd.0000129 PMC2217672 18235849

[B9] Caragata E. P. Rances E. Hedges L. M. Gofton A. W. Johnson K. N. O’Neill S. L. (2013). Dietary cholesterol modulates pathogen blocking by *Wolbachia*. *PLoS Pathog.* 9:e1003459 10.1371/journal.ppat.1003459 PMC3694857 23825950

[B10] Carvalho D. O. McKemey A. R. Garziera L. Lacroix R. Donnelly C. A. Alphey L. (2015). Suppression of a field population of aedes aegypti in brazil by sustained release of transgenic male mosquitoes. *PLoS Negl. Trop. Dis.* 9:e0003864 10.1371/journal.pntd.0003864 PMC4489809 26135160

[B11] Dong Y. Aguilar R. Xi Z. Warr E. Mongin E. Dimopoulos G. (2006). *Anopheles gambiae* immune responses to human and rodent *Plasmodium* parasite species. *PLoS Pathog.* 2:e52 10.1371/journal.ppat.0020052 PMC1475661 16789837

[B12] Dutra H. L. Rocha M. N. Dias F. B. Mansur S. B. Caragata E. P. Moreira L. A. (2016). *Wolbachia* blocks currently circulating zika virus isolates in Brazilian *Aedes aegypti* mosquitoes. *Cell Host Microbe* 19 771–774. 10.1016/j.chom.2016.04.021 27156023 PMC4906366

[B13] Fraiture M. Baxter R. H. Steinert S. Chelliah Y. Frolet C. Quispe-Tintaya W. (2009). Two mosquito LRR proteins function as complement control factors in the TEP1-mediated killing of *Plasmodium*. *Cell Host Microbe* 5 273–284. 10.1016/j.chom.2009.01.005 19286136

[B14] Freyvogel T. A. Staeubli W. (1965). The formation of the peritrophic membrane in culicidae. *Acta Trop.* 22 118–147. 14319771

[B15] Frolet C. Thoma M. Blandin S. Hoffmann J. A. Levashina E. A. (2006). Boosting NF-kappaB-dependent basal immunity of *Anopheles gambiae* aborts development of *Plasmodium berghei*. *Immunity* 25 677–685. 10.1016/j.immuni.2006.08.019 17045818

[B16] Hilgenboecker K. Hammerstein P. Schlattmann P. Telschow A. Werren J. H. (2008). How many species are infected with *Wolbachia*?–A statistical analysis of current data. *FEMS Microbiol. Lett.* 281 215–220. 10.1111/j.1574-6968.2008.01110.x 18312577 PMC2327208

[B17] Hoffmann A. A. Montgomery B. L. Popovici J. Iturbe-Ormaetxe I. Johnson P. H. Muzzi F. (2011). Successful establishment of *Wolbachia* in *Aedes* populations to suppress dengue transmission. *Nature* 476 454–457. 10.1038/nature10356 21866160

[B18] Hughes G. L. Koga R. Xue P. Fukatsu T. Rasgon J. L. (2011). *Wolbachia* infections are virulent and inhibit the human malaria parasite *Plasmodium falciparum* in anopheles gambiae. *PLoS Pathog.* 7:e1002043 10.1371/journal.ppat.1002043 PMC3098226 21625582

[B19] Hughes G. L. Rivero A. Rasgon J. L. (2014). *Wolbachia* can enhance *Plasmodium* infection in mosquitoes: implications for malaria control? *PLoS Pathog.* 10:e1004182 10.1371/journal.ppat.1004182 PMC4154766 25187984

[B20] Hughes G. L. Vega-Rodriguez J. Xue P. Rasgon J. L. (2012). *Wolbachia* strain wAlbB enhances infection by the rodent malaria parasite *Plasmodium berghei* in *Anopheles gambiae* mosquitoes. *Appl. Environ. Microbiol.* 78 1491–1495. 10.1128/AEM.06751-11 22210220 PMC3294472

[B21] Ito J. Ghosh A. Moreira L. A. Wimmer E. A. Jacobs-Lorena M. (2002). Transgenic anopheline mosquitoes impaired in transmission of a malaria parasite. *Nature* 417 452–455. 10.1038/417452a 12024215

[B22] Jiang X. Peery A. Hall A. B. Sharma A. Chen X. G. Waterhouse R. M. (2014). Genome analysis of a major urban malaria vector mosquito. *Anopheles stephensi.* *Genome Biol.* 15 459 10.1186/s13059-014-0459-2 PMC4195908 25244985

[B23] Joshi D. McFadden M. J. Bevins D. Zhang F. Xi Z. (2014). *Wolbachia* strain wAlbB confers both fitness costs and benefit on *Anopheles stephensi*. *Parasit Vectors* 7:336 10.1186/1756-3305-7-336 PMC4223616 25041943

[B24] Kambris Z. Blagborough A. M. Pinto S. B. Blagrove M. S. Godfray H. C. Sinden R. E. (2010). *Wolbachia* stimulates immune gene expression and inhibits plasmodium development in *Anopheles gambiae*. *PLoS Pathog.* 6:e1001143 10.1371/journal.ppat.1001143 PMC2951381 20949079

[B25] Killeen G. F. Barillas-Mury C. Thomas M. B. Greenwood B. (2013). Modulating malaria with *Wolbachia*. *Nat. Med.* 19 974–975. 10.1038/nm.3298 23921743

[B26] Kittayapong P. Baisley K. J. Baimai V. O’Neill S. L. (2000). Distribution and diversity of *Wolbachia* infections in Southeast Asian mosquitoes (Diptera: Culicidae). *J. Med. Entomol.* 37 340–345. 10.1093/jmedent/37.3.340 15535575

[B27] Kokoza V. Ahmed A. Woon Shin S. Okafor N. Zou Z. Raikhel A. S. (2010). Blocking of *Plasmodium* transmission by cooperative action of Cecropin A and Defensin A in transgenic *Aedes aegypti* mosquitoes. *Proc. Natl. Acad. Sci. U.S.A.* 107 8111–8116. 10.1073/pnas.1003056107 20385844 PMC2889521

[B28] Kumar S. Christophides G. K. Cantera R. Charles B. Han Y. S. Meister S. (2003). The role of reactive oxygen species on *Plasmodium* melanotic encapsulation in *Anopheles gambiae*. *Proc. Natl. Acad. Sci. U.S.A.* 100 14139–14144. 10.1073/pnas.2036262100 14623973 PMC283559

[B29] Kumar S. Molina-Cruz A. Gupta L. Rodrigues J. Barillas-Mury C. (2010). A peroxidase/dual oxidase system modulates midgut epithelial immunity in *Anopheles gambiae*. *Science* 327 1644–1648. 10.1126/science.1184008 20223948 PMC3510679

[B30] Laven H. (1967). Eradication of *Culex pipiens* fatigans through cytoplasmic incompatibility. *Nature* 216 383–384. 10.1038/216383a0 4228275

[B31] Livak K. J. Schmittgen T. D. (2001). Analysis of relative gene expression data using real-time quantitative PCR and the 2(-Delta Delta C(T)) Method. *Methods* 25 402–408. 10.1006/meth.2001.1262 11846609

[B32] Mains J. W. Brelsfoard C. L. Rose R. I. Dobson S. L. (2016). Female adult *Aedes albopictus* suppression by *Wolbachia*-infected male mosquitoes. *Sci. Rep.* 6:33846 10.1038/srep33846 PMC5034338 27659038

[B33] Moreira L. A. Iturbe-Ormaetxe I. Jeffery J. A. Lu G. Pyke A. T. Hedges L. M. (2009). A *Wolbachia* symbiont in *Aedes aegypti* limits infection with dengue, Chikungunya, and *Plasmodium*. *Cell* 139 1268–1278. 10.1016/j.cell.2009.11.042 20064373

[B34] Murdock C. C. Blanford S. Hughes G. L. Rasgon J. L. Thomas M. B. (2014). Temperature alters *Plasmodium* blocking by *Wolbachia*. *Sci. Rep.* 4:3932 10.1038/srep03932 PMC3909897 24488176

[B35] Pan X. Zhou G. Wu J. Bian G. Lu P. Raikhel A. S. (2012). *Wolbachia* induces reactive oxygen species (ROS)-dependent activation of the Toll pathway to control dengue virus in the mosquito *Aedes aegypti*. *Proc. Natl. Acad. Sci. U.S.A.* 109 E23–E31. 10.1073/pnas.1116932108 22123956 PMC3252928

[B36] Pinto S. B. Mariconti M. Bazzocchi C. Bandi C. Sinkins S. P. (2012). *Wolbachia* surface protein induces innate immune responses in mosquito cells. *BMC Microbiol.* 12(Suppl. 1):S11 10.1186/1471-2180-12-S1-S11 PMC3287508 22375833

[B37] Povelones M. Waterhouse R. M. Kafatos F. C. Christophides G. K. (2009). Leucine-rich repeat protein complex activates mosquito complement in defense against *Plasmodium* parasites. *Science* 324 258–261. 10.1126/science.1171400 19264986 PMC2790318

[B38] Ricci I. Cancrini G. Gabrielli S. D’Amelio S. Favi G. (2002). Searching for *Wolbachia* (Rickettsiales: Rickettsiaceae) in mosquitoes (Diptera: Culicidae): large polymerase chain reaction survey and new identifications. *J. Med. Entomol.* 39 562–567. 10.1603/0022-2585-39.4.562 12144285

[B39] Shahabuddin M. Fields I. Bulet P. Hoffmann J. A. Miller L. H. (1998). *Plasmodium gallinaceum*: differential killing of some mosquito stages of the parasite by insect defensin. *Exp. Parasitol.* 89 103–112. 10.1006/expr.1998.4212 9603495

[B40] Sinden R. E. Alavi Y. Raine J. D. (2004). Mosquito–malaria interactions: a reappraisal of the concepts of susceptibility and refractoriness. *Insect Biochem. Mol. Biol.* 34 625–629. 10.1016/j.ibmb.2004.03.015 15242703

[B41] Sinkins S. P. (2004). *Wolbachia* and cytoplasmic incompatibility in mosquitoes. *Insect Biochem. Mol. Biol.* 34 723–729. 10.1016/j.ibmb.2004.03.025 15242714

[B42] The malERA Consultative Group on Vector Control (2011). A research agenda for malaria eradication: vector control. *PLoS Med.* 8:e1000401 10.1371/journal.pmed.1000401 PMC3026704 21311587

[B43] Werren J. H. Baldo L. Clark M. E. (2008). *Wolbachia*: master manipulators of invertebrate biology. *Nat. Rev. Microbiol.* 6 741–751. 10.1038/nrmicro1969 18794912

[B44] WHO (2015). *World Malaria Report 2015*. Geneva: WHO Press.

[B45] Xi Z. Joshi D. (2015). “Genetic control of malaria and dengue using *Wolbachia*,” in *Genetic Control of Malaria and Dengue*, ed. Adelman Z. N. (Amsterdam: Elsevier Inc), 305–333.

[B46] Zele F. Nicot A. Berthomieu A. Weill M. Duron O. Rivero A. (2014). *Wolbachia* increases susceptibility to *Plasmodium* infection in a natural system. *Proc. Biol. Sci.* 281:20132837 10.1098/rspb.2013.2837 PMC3924077 24500167

[B47] Zug R. Hammerstein P. (2012). Still a host of hosts for *Wolbachia*: analysis of recent data suggests that 40% of terrestrial arthropod species are infected. *PLoS ONE* 7:e38544 10.1371/journal.pone.0038544 PMC3369835 22685581

