# Supplementary material for: The Maternally Inheritable Wolbachia wAlbB Induces Refractoriness to Plasmodium berghei in Anopheles stephensi
Source: Front Microbiol. 2017 Mar 8;8:366. doi: 10.3389/fmicb.2017.00366 (PMC5340780; doi:10.3389/fmicb.2017.00366)
Supplement: Supplementary file 1 [file Table_1.PDF]

Table S1: The sequence of primers used to measure the gene expression by real-time PCR

Gene name	Gene ID	Primer name	Primer sequences
TEP-1	ASTE010227	TEP1 F	AATTACCATCGACGGACAGC
		TEP1 R	CACGGATCGTCACATGAATC
REL-1	ASTE011378	REL-1 F	AAACGTTCCCCAGCATACAG
		REL-1 R	CCTCGACGTCCTTCTTCTTG
PGRP-LC	ASTE002618	PGRP-LC F	GCGATACCGAGTGTGAGGAT
		PGRP-LC R	GTGATGTCGGACGAGTTGTG
DEF-1	ASTE011281	DEF-1 F	AGTCGTGGTCCTGGCGGCTCT
		DEF-1 R	ACGAGCGATGCAATGCGCGGCA
LRIM-1	ASTE000814	LRIM 1 F	AAGCGCAAGGAGTATGCACT
		LRIM-1 R	CGGTAGCTTCCTTTCCAATG
CAT-1	ASTE010206	CAT1 F	GGA CTT CAT TTC GCT TCG AC
		CAT1 R	CTT GCA GTA CAC TGG CTT GC
RPS6	ASTE007172	RPS6 F	ACGACCACAAGCTGCGTCAC
		RPS6 R	GTCAGCACACCCTGCTTCATG
