# Supplementary material for: The Maternally Inheritable Wolbachia wAlbB Induces Refractoriness to Plasmodium berghei in Anopheles stephensi
Source: Front Microbiol. 2017 Mar 8;8:366. doi: 10.3389/fmicb.2017.00366 (PMC5340780; doi:10.3389/fmicb.2017.00366)
Supplement: Supplementary file 2 [file Table_2.PDF]

Table S2: Ooycsts and sporozoites in wild-type LIS, LB1 or LBT mosquitoes. *Pb, Plasmodium berghei.*

**Fig. 1A (*Pb* oocysts)**	**LIS**	**LB1**
Average parasitemia (%) in mice used for feeding (range)	16.87%(15.39-18.45)	
Average exflagellations per field in mice (range)	0.12 (-)	
Number of samples for midgut oocyst (n)	41	35
Range (Min. -Max.)	0-261	0-73
Median	36	16
Mean	47.02	23.74
Prevalence rate (%)	90.48	80.00
Passed normality test (*=0.05)?	No	Yes
Mann-Whitney test (p value) -LIS vs LB1		0.0219

-=missed recording

**Fig. 1B (*Pb* oocysts)**	**LIS**	**LB1**
Average parasitemia (%) in mice used for feeding (range)	21% (4-33)	
Average exflagellations in mice used for feeding (range)	0.45(0.05-1.27)	
Number of samples for midgut oocyst (n)	154	91
Range (Min. -Max.)	0-732	0-602
Median	67.5	25
Mean	123.9	94.58
Prevalence	90.26	76.92
Passed normality test (*=0.05)?	No	No
Mann-Whitney test (p value) -LIS vs LB1		0.0044

**Fig. 1C (*Pb* oocysts)**	**LBT**	**LB1**
Average Parasitemia (%) in mice used for feeding (range)	24%(8-46)	
Average exflagellations per field in mice (range)	1.16 (0—2.8)	
Number of samples for midgut oocysts (n)	116	86
Range (Min. -Max.)	0-733	0-650
Median	73	34

Mean	126.8	104.1
Prevalence	93.96	86.05
Passed normality test (*=0.05)?	No	No
Mann-Whitney test (p value) -LBT vs LB1		0.0432

**Fig. 2A (*Pb* sporozoites)**	**LIS**	**LB1**
Average Parasitemia (%) in mice used for feeding (range)	8.84(5.67-14.6)	
Average exflagellations per field in mice (range)	0.06(0-0.3)	
Number of samples for salivary gland sporozoites (n)	43	32
Range (Min. -Max.)	0-29,300	0-6,500
Median	900	75
Mean	3586	931.1
Prevalence rate (%)	74.42	59.38
Passed normality test (*=0.05)?	No	No
Mann-Whitney test (p value) -LIS vs LB1		0.0136

**Fig. 2B (*Pb* sporozoites)**	**LIS**	**LB1**
Average Parasitemia (%) in mice used for feeding (range)	3%(1-5%)	
Average exflagellations in mice used for feeding (range)	0.45(0-5)	
Number of samples for salivary gland sporozoites(n)	64	59
Range (Min. -Max.)	0-38,400	0-112,650
Median	2,865	1,140
Mean	7,975	6,891
Prevalence	87.50	72.88
Passed normality test (*=0.05)?	No	No
Mann-Whitney test (p value) - LIS vs LB1		0.0426

**Fig. 2C (*Pb* sporozoites)**	**LBT**	**LB1**
Average Parasitemia in mice used for feeding (range)	4(1.05-4.75)	
Average exflagellations per field in mice (range)	0.31 (0-2)	
Number of samples for salivary gland sporozoites (n)	36	23
Range (Min. -Max.)	0-75,675	0-58,800

Median	1463	120
Mean	7,638	6,670
Prevalence (%)	94.44	60.87
Passed normality test (*=0.05)?	No	No
Mann-Whitney test (p value) -LBT vs LB1		0.0115
